# Overweight/Obesity and Body Fat Percentage in Relation to Iron Status Among Indonesian Women of Reproductive Age: Evidence from a Case–Control Study

**DOI:** 10.3390/nu18162583

**Published:** 2026-08-07

**Authors:** Qonita Rachmah, Hai Phung, Faruk Ahmed

**Affiliations:** 1 Public Health, School of Medicine and Dentistry, Griffith University, Gold Coast, QLD 4215, Australia; qonita.rachmah@griffithuni.edu.au (Q.R.); hai.n.phung@griffith.edu.au (H.P.); 2 Department of Nutrition, Faculty of Public Health, Universitas Airlangga, Surabaya 60115, Indonesia

**Keywords:** anaemia, iron deficiency anaemia, obesity, hepcidin, inflammation, women of reproductive age, Indonesia

## Abstract

**Background/Objectives:** Obesity-related inflammation may disrupt iron homeostasis and increase the risk of iron deficiency, yet evidence among women in low- and middle-income countries remains limited. This study examined the independent association between overweight/obesity and iron status among Indonesian women of reproductive age. **Methods**: A case–control study was conducted among 340 nonpregnant women aged 20–29 years in Surabaya, Indonesia. Sociodemographic, dietary, menstrual, and anthropometric data were collected alongside venous blood samples. Logistic regression models were used to examine associations between adiposity measures and iron deficiency (ID), anaemia, and iron deficiency anaemia (IDA), adjusting for potential confounders. **Results**: Inflammation-adjusted serum ferritin was significantly higher among women with overweight/obesity than among normal-weight women (22.39 ± 21.7 vs. 17.78 ± 17.6 µg/L; *p* = 0.033). Consistent with this, the prevalence of ID was lower among women with overweight/obesity, likely reflecting inflammation-related ferritin elevation that may influence diagnostic classification. BMI-defined overweight/obesity was not independently associated with ID, anaemia, or IDA. In contrast, higher body fat percentage was inversely associated with ID (AOR 0.93; 95% CI 0.86, 0.99) and IDA (AOR 0.86; 95% CI 0.75, 0.99). Elevated alpha-1-acid glycoprotein (AGP) was independently associated with higher odds of IDA, and menstrual iron loss was an independent predictor of anaemia and IDA. **Conclusions**: BMI alone is insufficient to capture the relationship between adiposity and iron status in women of reproductive age. Body fat percentage appears to be a more sensitive indicator of adiposity-related iron risk. These findings suggest that inflammation associated with adiposity may alter iron biomarkers, and reliance on BMI alone may obscure the true burden of iron deficiency in this population.

## 1. Introduction

Iron deficiency and obesity are among the most prevalent nutritional burdens affecting women globally, particularly in low- and middle-income countries (LMICs) [1,2]. Growing evidence suggests these conditions may be interrelated [3,4]. Iron deficiency is the leading cause of anaemia, a condition in which haemoglobin levels are insufficient to transport adequate oxygen to the body’s tissues, affecting 30.5% of non-pregnant women globally, with the highest burden reported in the South-East Asia Region (46.4%) [2]. Concurrently, 44% of adult women worldwide are living with overweight or obesity, a prevalence slightly higher than that observed among men [5]. The coexistence of these conditions, both at the population level and within individuals, raises concerns that obesity-related metabolic disturbances may negatively affect iron status [6,7].

The most widely proposed mechanism linking overweight/obesity to impaired iron status is chronic low-grade systemic inflammation. Obesity-related inflammation stimulates the synthesis of hepcidin, a key iron-regulatory hormone, which suppresses intestinal iron absorption and reduces bioavailability [8]. Despite this plausible biological pathway, relatively few studies have directly measured hepcidin concentrations, limiting understanding of inflammation-mediated iron dysregulation among women with overweight/obesity. Elevated concentrations of inflammatory markers such as C-reactive protein (CRP) have consistently been observed among women with obesity compared with those of normal-weight women [3,4,9].

Empirical findings on the association between overweight/obesity and iron status, however, remain inconsistent. Several studies have reported lower serum iron and transferrin saturation, alongside elevated ferritin concentrations, among women with obesity, suggesting inflammation-driven iron sequestration [3,9,10]. In contrast, other studies have found no significant association between overweight/obesity and iron biomarkers [11,12,13]. A major methodological limitation is that ferritin, a widely used biomarker of iron status, increases during inflammation and may therefore overestimate iron stores in individuals with overweight and obesity. Despite recommendations to adjust ferritin concentrations for inflammation [14], many studies continue to rely on unadjusted values [3,15,16], potentially obscuring the relationship between adiposity and iron deficiency. Additionally, dietary factors may influence the relationship between obesity and iron status. Obesity associated diets are often energy-dense yet low in bioavailable iron, representing a potential pathway to iron deficiency independent of inflammation. However, this mechanism has rarely been disentangled from inflammation-related pathways in previous studies [17]. Furthermore, most existing evidence comes from high-income countries, despite the growing burden of overweight and obesity in LMICs and studies often lack comparisons with normal-weight populations.

Indonesia, an LMIC country undergoing rapid nutritional transition, provides an important setting for examining this relationship between adiposity and iron status. Anaemia affects 27.2% of nonpregnant women, with the highest prevalence among those aged 15–24 years, while 44.4% of women have overweight or obesity [18]. Despite the coexistence of these conditions, their interrelationship remains poorly understood. To our knowledge, this is the first study in an LMIC setting to concurrently assess inflammation-adjusted ferritin and hepcidin while accounting for dietary iron intake, as well as iron absorption enhancers and inhibitors as covariates. Therefore, this study aimed to investigate the association between overweight/obesity and iron status among Indonesian nonpregnant women of reproductive age. Secondary objectives were to examine the potential roles of systemic inflammation and dietary factors in this association. The findings may provide insight into the influence of obesity-related inflammation on iron biomarkers and inform integrated nutrition strategies targeting women in Indonesia.

## 2. Materials and Methods

### 2.1. Study Design and Participants

This case–control study was conducted among nonpregnant, non-lactating women aged 20–29 years residing in Surabaya, East Java, Indonesia. This age range was selected to minimise heterogeneity related to age-related physiological factors that may affect iron biomarkers and confound the association between adiposity and iron status. In addition, this age group was selected due to the high burden of anaemia among women of reproductive age reported in the Indonesia Basic Health Research Report 2019 [18]. Eligible participants had no history of menstrual abnormalities (e.g., amenorrhoea, polymenorrhoea). Women were excluded if they had medical conditions (i.e., diabetes, kidney disease, liver disease, and haematological disorders) or were taking medications, including iron supplements, that could affect body weight, iron status, or inflammation.

### 2.2. Sample Size

The sample size was calculated to detect a difference in the prevalence of iron deficiency between women with overweight/obesity and normal-weight women, with a two-sided significance level of 5% and 80% statistical power. Based on previous studies reporting iron deficiency prevalence of approximately 12–13% among normal-weight women and 20–25% among women with obesity [9,19,20], the sample size calculation assumed a 13-percentage-point difference in prevalence (12% vs. 25%) as the expected effect size. As population-specific data for Indonesia were unavailable, prevalence estimates from international studies were used. Using standard formulas for comparing two independent proportions with a continuity correction and a 1:1 ratio, a minimum of approximately 150 participants per group was required [21].

### 2.3. Recruitment and Screening

Participants were recruited using community-based convenience sampling within a university setting, targeting female students and staff. Recruitment strategies included printed posters displayed across campus facilities and broadcast messages disseminated via university-affiliated social media platforms. Recruitment materials outlined the study objectives, eligibility criteria, procedures, data-collection site, and benefits of participation, including complimentary health-screening results.

A total of 632 women were screened based on their body mass index (BMI), with those having a BMI < 18.5 kg/m^2^ being excluded. Participants were categorised into the case group (women with overweight/obesity, BMI ≥ 23 kg/m^2^) or the control group (normal-weight, BMI 18.5–22.9 kg/m^2^) using Asian-specific BMI cut-offs [22]. Frequency matching was applied to ensure comparable distributions of age, occupation, and educational attainment between groups. Written informed consent was obtained from all participants before study enrolment. Of 623 women screened, 341 met the inclusion criteria and consented; one was excluded due to inability to provide a blood sample, yielding a final sample of 340 women (174 cases and 166 controls).

### 2.4. Data Collection

Eligible participants attended a scheduled appointment at the Nutrition Laboratory, Universitas Airlangga, Surabaya, for data and blood specimen collection. Data were collected using an interviewer-administered structured questionnaire to obtain information on sociodemographic characteristics (age, residence, education, occupation, and income), general morbidity (self-reported acute health complaints within the preceding two weeks), physical activity assessed using the validated International Physical Activity Questionnaire (IPAQ) [23,24], and menstrual history (age at menarche, cycle regularity, duration, and flow).

Dietary iron intake was assessed using a 60-item semi-quantitative food frequency questionnaire (SQFFQ) developed for Indonesian women of reproductive age. The SQFFQ captured dietary iron intake as well as key modulators of iron bioavailability, including vitamin C (enhancer) and zinc, calcium, phytate, and fibre (inhibitors). The questionnaire was validated against 3-day food records, with agreement assessed using Bland–Altman analysis. Reported food consumption frequencies and portion sizes were converted to estimated daily nutrient intake using the Indonesian Food Composition Table (TKPI) and USDA food composition databases via Nutrisurvey 2007 software (University of Hohenheim, EBISpro, Willstätt, Germany and SEAMEO RECFON, Jakarta, Indonesia). Height (to the nearest 0.1 cm) was measured without shoes using a portable stadiometer (SECA 213; seca GmbH & Co. KG, Hamburg, Germany), while weight (to the nearest 0.1 kg) and body fat percentage were measured using a bioelectrical impedance analyser (OMRON HBF-375; OMRON Corporation, Kyoto, Japan). Waist circumference (to the nearest 0.1 cm) was measured using a non-stretchable measuring tape (SECA 201; seca GmbH & Co. KG, Hamburg, Germany) at the midpoint between the lowest rib and the iliac crest [25]. Venous blood samples were collected in the morning by trained phlebotomists using standard aseptic techniques. Blood was drawn into two tubes per participant: one EDTA tube (~3 mL) for whole blood analysis of haematological parameters, and one plain serum-separator tube (SST, ~5 mL) for serum separation and biochemical analyses. All samples were appropriately labelled, processed and stored in accordance with established laboratory protocols prior to biochemical analysis.

### 2.5. Biochemical Analysis

Haemoglobin (Hb), serum ferritin, C-reactive protein (CRP), α1-acid glycoprotein (AGP), and hepcidin were analysed at a nationally accredited commercial diagnostic laboratory. A whole-blood sample was used to measure haematological parameters (Hb, haematocrit [Hct], and erythrocyte count) with an automated haematology analyser (Mindray BC-2800 series, Mindray Bio-Medical Electronics Co., Ltd., Guangdong, China), following routine calibration and quality control procedures to ensure analytical accuracy and precision. Serum ferritin was measured using a blood chemistry analyser (ICHEM-I UBIO 535, UBIO Biotechnology Systems Pvt. Ltd., New Delhi, India), with a reported repeatability coefficient of variation (CV) < 1%. CRP, AGP, and hepcidin concentrations were determined using commercially available ELISA kits according to the manufacturers’ instructions. Specifically, AGP was measured using the Human Alpha-1B-Glycoprotein (A1BG) ELISA Kit (Catalogue No. E1430Hu, Korain Biotech, Shanghai, China), with intra- and inter-assay coefficients of variation (CVs) of 4.87% and <10%, respectively. CRP was measured using the AccuDiag™ C-Reactive Protein ELISA Kit (Catalogue No. 1668-P1, Diagnostic Automation/Cortez Diagnostics Inc., Woodland Hills, CA, USA), with a reported CV of <1%. Serum hepcidin was assessed using the Human Hepcidin (HEPC) ELISA Kit (Catalogue No. E1019Hu, Korain Biotech, China), with intra- and inter-assay CVs of 4.40% and <10%, respectively. All ELISA assays were performed in duplicate, with assay validity confirmed using standards and controls. Absorbance was measured using a Diatek DR-200Bc microplate reader (Diatek Instruments, Jiangsu, China). AGP value was converted from mg/dL to g/L [14]. As serum ferritin is an acute-phase reactant influenced by inflammation, concentrations were adjusted for inflammatory status using CRP and AGP values, following the regression correction approach [14,26,27]. Iron deficiency (ID) was defined as inflammation-adjusted ferritin concentrations < 15 µg/L [26]. Anaemia was defined as haemoglobin < 12 g/dL, and iron deficiency anaemia (IDA) as the presence of both anaemia and ID. Elevated CRP was defined as >5 mg/L and AGP > 1 g/L [28]. High hepcidin concentrations were defined as values above the median.

### 2.6. Statistical Analyses

Statistical analyses were performed using IBM SPSS Statistics 31 for Windows (IBM Corporation, Armonk, NY, USA). Data are presented as percentages, mean ± standard deviation (SD) or median (interquartile range, IQR). Normality was assessed using the Kolmogorov–Smirnov test and visual inspection of histograms. Skewed variables (CRP, AGP, and hepcidin) were analysed using non-parametric methods. Differences between case and control groups in sociodemographic characteristics, morbidity, dietary intake, physical activity, menstrual history, and biochemical markers were assessed using chi-square tests for categorical variables and independent *t*-tests or Mann–Whitney U tests for continuous variables, as appropriate. Binary logistic regression analyses were conducted to examine the associations between adiposity indicators and (1) ID, (2) anaemia, and (3) IDA.

Bivariate analyses were initially performed to assess crude associations (Supplementary Table S2). Variables associated with each outcome at *p* < 0.25 were considered for multivariable models. Covariates were selected a priori based on biological plausibility to account for confounding and to assess inflammation-related pathways. Multivariable models were constructed sequentially, based on conceptual relevance, to evaluate the stability of associations [29]. Multicollinearity was assessed using the variance inflation factor (VIF), with all values below 5 indicating no significant collinearity. Results are presented as adjusted odds ratios (AORs) with 95% confidence intervals (CIs), and statistical significance was determined accordingly. The final models included all selected covariates for each outcome.

## 3. Results

A summary of participant characteristics is presented in Table 1. Of the participants, 166 (48.8%) had a normal BMI, while 174 (51.2%) had overweight or obesity (18.8% overweight and 32.4% obesity). Sociodemographic characteristics were largely comparable between groups, with no significant differences observed in age, residential place, education, or employment. However, the proportion of women with higher monthly income was significantly greater among those with overweight/obesity (*p* = 0.009). Anthropometrically, BMI, body fat percentage, and waist circumference were all significantly higher in the group with overweight/obesity (all *p* < 0.001), as expected. Regarding lifestyle and reproductive characteristics, physical activity differed by case and control groups: women with overweight/obesity reported higher Metabolic Equivalents of Task (MET) scores and a greater prevalence of moderate-to-vigorous-intensity activity (*p* = 0.004). Age at menarche was significantly lower among women with overweight/obesity (*p* = 0.005), while estimated menstrual blood loss and iron loss were similar between groups.

Dietary characteristics of the participants are presented in Supplementary Table S1. There were no significant differences in total energy intake or intake of vitamin C, zinc, calcium, phytate, and fibre between groups. Similarly, no differences were observed in the frequency of tea, coffee, or animal-source food consumption, or in dietary diversity. Although mean iron intake (mg/day) did not differ between groups (*p* = 0.928), its distribution across tertiles varied significantly (*p* = 0.040), with women with overweight/obesity concentrated in the middle tertile.

Mean haemoglobin concentrations were similar between groups (12.6 g/dL in normal-weight and 12.8 g/dL in women with overweight/obesity; *p* = 0.146). Conversely, inflammation-adjusted serum ferritin was significantly higher in women with overweight/obesity (22.39 ± 21.7 µg/L) compared with normal-weight women (17.78 ± 17.6 µg/L; *p* = 0.033). No significant differences were observed in CRP, AGP, or hepcidin concentrations between groups (Table 2).

**Figure 1:**
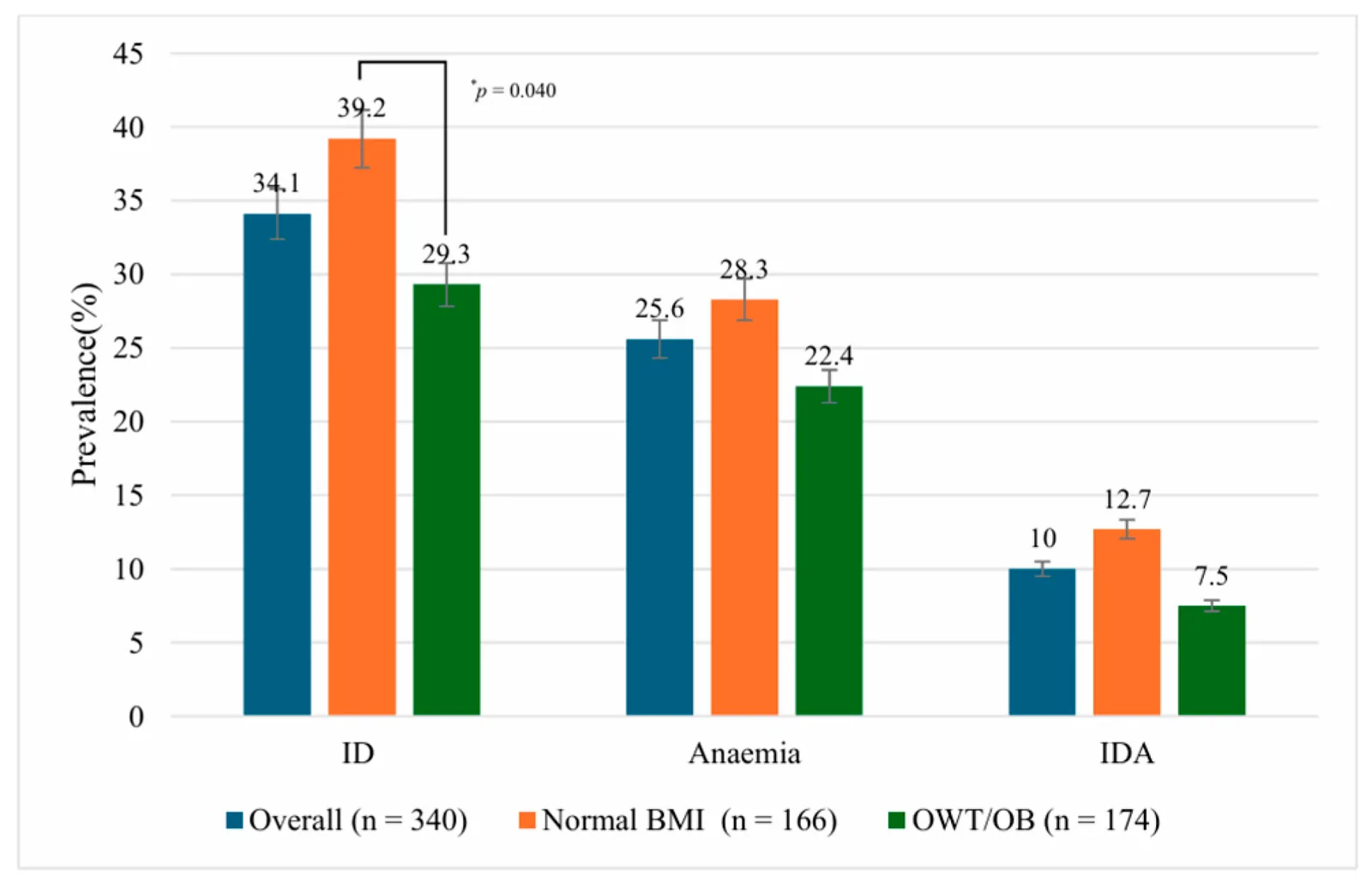
Prevalence of ID, anaemia, and IDA among the normal-weight group and the group with overweight/obesity. Bars represent the percentage of participants in each BMI group who meet the respective diagnostic criteria. Error bars indicate 95% CI. * *p* = 0.040 between normal-weight and OWT/OB in the ID column; no other significant differences were observed.

**Figure 2:**
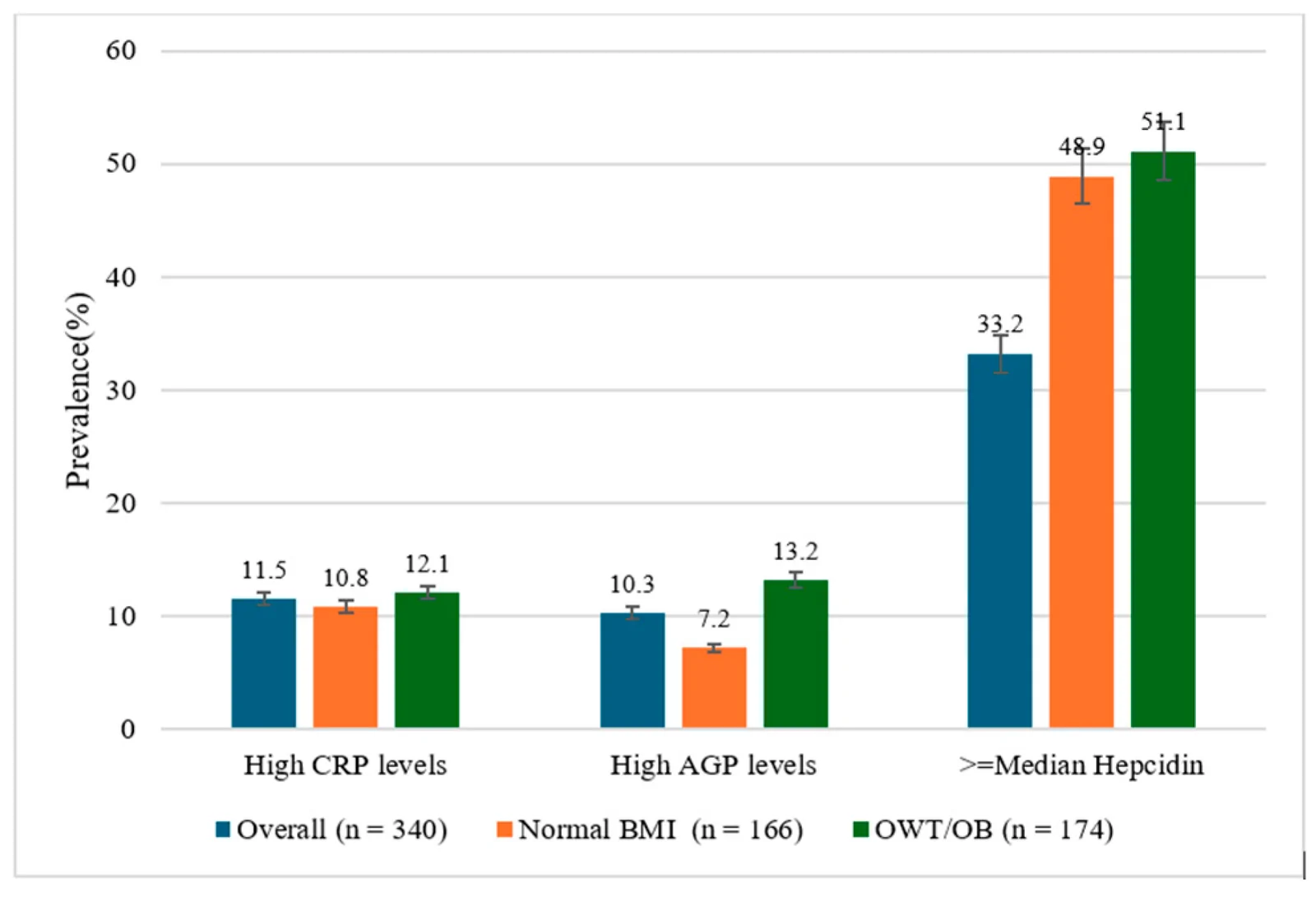
Prevalence of inflammatory (CRP and AGP) and hepcidin categories among the normal-weight group and the group with overweight/obesity. Bars represent the percentage of participants within each BMI group. Error bars indicate 95% CI.

Figure 1 illustrates the prevalence of ID, anaemia, and IDA by BMI group. ID was significantly less prevalent among women with overweight/obesity than among normal-weight women (*p* = 0.040). Anaemia and IDA were also less common in women with overweight/obesity, although these differences were not statistically significant. Figure 2 shows the distribution of inflammatory and iron-regulatory biomarker categories by BMI group. Elevated CRP, AGP, and hepcidin concentrations were more frequent in women with overweight/obesity, although these differences were not statistically significant (*p* = 0.737, *p* = 0.076, and *p* = 1.000, respectively).

### 3.1. Iron Deficiency (ID)

Multivariable logistic regression models for ID are presented in Table 3. In the crude analysis, overweight/obesity was not significantly associated with ID (OR 0.64; 95% CI 0.41, 1.01; *p* = 0.056). After adjustment for socio-demographic factors (Model I), the estimate remained unchanged. With the further addition of dietary variables in Model II, overweight/obesity was inversely and significantly associated with ID (AOR 0.58; 95% CI 0.36, 0.95; *p* = 0.029). In the same model, low-intensity physical activity was associated with higher odds of ID compared with moderate-to-vigorous activity (AOR 1.65; 95% CI 1.01, 2.71; *p* < 0.05), although this association was attenuated and did not remain significant in the final model. In the fully adjusted model (Model III), the association between overweight/obesity and ID attenuated and was no longer statistically significant (AOR 0.82; 95% CI 0.43, 1.56), suggesting that body fat percentage, menstrual iron loss, and hepcidin concentrations account for part of the earlier association. Body fat percentage was the only predictor independently and inversely associated with ID in the final model (AOR 0.93; 95% CI 0.86, 0.99; *p* = 0.046). No other socio-demographic, dietary, or biomarker variables were independently associated with ID in the fully adjusted model. Model fit improved, with Nagelkerke R^2^ increasing from 0.015 in the crude model to 0.122 in Model III.

### 3.2. Anaemia

Multivariable logistic regression models for anaemia are presented in Table 4. Overweight/obesity was not associated with anaemia in either the crude (OR 0.71; 95% CI 0.44, 1.16) or fully adjusted model (AOR 1.07; 95% CI 0.54, 2.11). Dietary intakes of iron, zinc, and phytate did not reach statistical significance after adjustment (*p* > 0.05). Menstrual iron loss was the only variable independently and significantly associated with anaemia in the final model (AOR 1.01; 95% CI 1.00, 1.02; *p* = 0.003). Higher body fat percentage was inversely associated with anaemia, though this was not statistically significant (AOR 0.59; 95% CI 0.29, 1.18). No other dietary or biomarker variables were independently associated with anaemia in the fully adjusted model. Nagelkerke R^2^ increased from 0.008 in the crude model to 0.098 in Model II.

### 3.3. Iron Deficiency Anaemia (IDA)

Multivariable logistic regression models for IDA are presented in Table 5. Consistent with the anaemia findings, overweight/obesity was not associated with IDA in either the crude (OR 0.56; 95% CI 0.27, 1.15) or fully adjusted model (AOR 0.65; 95% CI 0.18, 2.32; *p* = 0.503). Body fat percentage was inversely associated with IDA in the final model (AOR 0.86; 95% CI 0.75, 0.99; *p* = 0.027), while menstrual iron loss was positively associated (AOR 1.02; 95% CI 1.00, 1.03; *p* = 0.008). Higher iron intake was positively associated with IDA (AOR 1.02; 95% CI 1.00, 1.04; *p* = 0.018). Elevated AGP was associated with higher odds of IDA in the final model (AOR 8.77; 95% CI 1.01, 77.34; *p* = 0.045), consistent with the role of chronic subclinical inflammation in impairing iron absorption.

However, the very wide confidence interval reflects low outcome prevalence and should be interpreted cautiously. Waist circumference, education, age at menarche, zinc, phytate, animal-source foods intake, physical activity, vitamin C, tea and coffee intake, and dietary diversity were not independently associated with IDA in the fully adjusted model. Nagelkerke R^2^ increased from 0.016 in the crude model to 0.265 in the final model.

## 4. Discussion

This case–control study provides insight into the association between overweight/obesity and iron status among young adult nonpregnant women in Indonesia. Several key findings emerged. First, inflammation-adjusted ferritin concentrations were significantly higher among women with overweight/obesity than among their normal-weight counterparts. Second, overweight/obesity, as defined by BMI, was not independently associated with iron deficiency (ID), anaemia, or iron deficiency anaemia (IDA). In contrast, body fat percentage was inversely associated with these iron status outcomes, suggesting that adiposity may be more relevant to iron status than BMI alone. Third, elevated AGP concentrations were independently associated with increased odds of IDA, highlighting the potential role of chronic inflammation in iron dysregulation.

Overall, the participants in the normal-weight group and the group with overweight/obesity had comparable sociodemographic characteristics and broadly similar dietary patterns. Although women with overweight/obesity in this study reported slightly more frequent consumption of animal-source food and marginally higher vitamin C intake, estimated daily iron intake and overall diet diversity did not differ meaningfully between groups. This relative homogeneity likely reflects a shared food environment and may explain the limited dietary variation observed between BMI categories [34,35,36]. These dietary variables were subsequently included in multivariate analyses, along with other covariates, to assess the independent association between overweight/obesity and iron status.

The bivariate analysis showed that haemoglobin concentrations did not differ significantly between groups, suggesting that any impairment in iron metabolism had not yet translated into measurable effects on erythropoiesis. However, women with overweight/obesity exhibited significantly higher adjusted serum ferritin concentrations than normal-weight women, which may reflect underlying low-grade inflammation rather than greater iron stores [37,38]. Similar findings have been reported among women with obesity, in whom elevated ferritin concentrations coexist despite evidence of impaired iron metabolism [3,4,20,39]. In the present study, although ferritin values were adjusted for CRP and AGP, elevated ferritin concentrations persisted, suggesting that conventional inflammatory markers may not fully capture adiposity-related alterations in iron regulation [37,40].

The inflammatory markers, CRP and AGP concentrations, did not differ significantly between the two groups. However, when participants were stratified into three BMI categories, AGP concentrations differed significantly across BMI categories. Post hoc analysis using the least significant difference (LSD) test showed that women with obesity had significantly higher AGP concentrations than women with normal weight (*p* = 0.006) and those with overweight (*p* = 0.028). This pattern suggests a dose–response relationship between adiposity and chronic inflammation. CRP demonstrated a similar trend but did not reach statistical significance. Mean hepcidin concentrations did not differ significantly between groups. Nevertheless, women with overweight/obesity in this study exhibited higher hepcidin concentrations than those reported in studies of otherwise healthy women with obesity [41,42]; although concentrations remained lower than those observed among women with morbid obesity [42,43]. Despite the absence of a significant group difference, this study noted a significant positive correlation between hepcidin and ferritin concentrations (r = 0.347, *p* = 0.006). Elevated hepcidin concentrations were observed among participants with higher ferritin concentrations, supporting the hypothesis that hepcidin upregulation may trigger iron sequestration [9,44].

The results further indicated a lower prevalence of ID among women with overweight/obesity. Although ferritin is widely used as an indicator of iron stores, it is also an acute-phase reactant; therefore, inflammation-related elevations may mask iron-store depletion and reduce the likelihood of meeting diagnostic thresholds for ID. Accordingly, the lower prevalence of ID observed in the present study may largely be attributable to the ferritin-based diagnosis of ID, as previously explained by Zhao et al. [45]. Moreover, ID accounted for only 33% of anaemia cases among women with overweight/obesity in this study, suggesting that ID alone did not fully explain the observed burden of anaemia [46]. Other factors, including inflammation-related alterations in iron metabolism, may also contribute to anaemia in this population.

Multivariate analyses revealed no significant association between overweight/obesity and ID, anaemia, or IDA, consistent with findings reported among healthy Vietnamese women [47] and female military personnel in the United States [10]. BMI-defined adiposity did not predict iron-related outcomes in this sample, likely because the degree of adiposity was insufficient to induce the level of inflammation necessary to trigger clinically meaningful hepcidin-mediated iron restriction [3,10]. Hepcidin is the key regulatory hormone governing systemic iron homeostasis; when upregulated, it degrades ferroportin, reduces intestinal iron absorption, and promotes iron sequestration within macrophages [48,49]. Evidence suggests that this pathway becomes clinically relevant at higher levels of adiposity, particularly among individuals with a BMI exceeding 35 kg/m^2^ and those with pre-existing morbidity [10,43]. In the present study, most women in the overweight/obesity group had a BMI below 30 kg/m^2^ (82.18%), which may explain the absence of a detectable adverse effect on iron status.

An interesting finding was the inverse association between body fat percentage and both ID and IDA, consistent with previous studies [10,15]. Compared with BMI, body fat percentage more directly reflects metabolically active adipose tissue and its associated inflammatory activity, and may vary substantially among individuals with the same BMI [50]. Increased adiposity (as indicated by higher body fat) may elevate ferritin concentrations through activation of the acute-phase response, while ferritin remains the primary biomarker used to diagnose ID [51]. However, in the absence of biomarkers that are less susceptible to inflammation, such as serum iron, transferrin saturation, or soluble transferrin receptor (sTfR), definitive confirmation of functional iron deficiency was not possible. It is plausible that women with higher adiposity and subclinical inflammation maintained ferritin concentrations above the diagnostic threshold despite compromised iron stores [4,9]. The findings for AGP in the multivariate model support this interpretation. Elevated AGP concentrations were independently associated with higher odds of IDA, suggesting a role for chronic low-grade inflammation in the pathways linking adiposity and iron status. Menstrual iron loss was also associated with anaemia and IDA, underscoring the importance of physiological iron loss as a determinant of iron status, independent of adiposity-related factors [52]. The positive association between dietary iron intake and IDA in the fully adjusted model likely reflects reverse causation; women experiencing symptoms of iron depletion may have increased their consumption of iron-rich foods, rather than dietary iron intake causing IDA. Consequently, this finding should be interpreted with caution. Collectively, these findings suggest that iron status among women of reproductive age is shaped by the interplay between inflammatory burden and physiological iron demand, two factors that are not adequately captured by BMI alone. This has a practical consequence: women with overweight/obesity who have near-normal BMI values and no overt signs of inflammation may still be at risk of iron depletion, particularly when menstrual losses are substantial.

A major strength of this study is its comprehensive assessment of adiposity and iron status, incorporating body fat percentage alongside inflammatory markers, dietary intake, and estimates of menstrual blood and iron loss. Unlike many previous studies that adjusted mainly for sociodemographic factors [17], this study simultaneously accounted for both dietary and menstrual determinants of iron status, enabling a more rigorous evaluation of the independent relationship between adiposity and iron status. The inclusion of a normal-weight comparison group further strengthens the design by allowing direct comparison of iron status across adiposity groups, an approach less commonly reported in prior studies [7]. Given the limited evidence from LMICs, where dietary patterns, body composition profiles, and inflammatory burdens differ substantially from high-income settings, these findings provide an important foundation for future research examining whether adiposity-related iron dysregulation contributes to the persistently high burden of anaemia in similar populations.

Several limitations should be acknowledged. First, the case–control design precludes causal inference and prevents the determination of the temporal direction of associations between adiposity, inflammation, and iron status. Second, the assessment of iron status was constrained by the absence of biomarkers less susceptible to acute-phase responses. Soluble transferrin receptor (sTfR), which reflects tissue-level iron deficiency independently of inflammatory status, was not measured. Inclusion of this biomarker would have enabled differentiation between true iron depletion and inflammation-driven elevation of ferritin concentrations [53]. The absence of transferrin saturation and serum iron measurements limited the evaluation of functional iron availability and the identification of potential iron sequestration. In addition, women with metabolic comorbidities were excluded because these conditions are often associated with distinct dietary patterns that may independently influence iron status and confound its association with adiposity. Excluding women with metabolic comorbidity, together with recruitment from a university setting, may limit the generalizability of the findings. Despite these limitations, this study provides evidence that adiposity-related inflammation may complicate the interpretation of ferritin-based indicators of iron status in nonpregnant women. These findings have important implications for the identification and management of ID in populations experiencing the dual burden of obesity and anaemia.

## 5. Conclusions

In conclusion, BMI-defined adiposity was not independently associated with iron status, despite higher inflammation-adjusted ferritin concentrations among participants with overweight or obesity. In contrast, body fat percentage was inversely associated with iron status-related outcomes. Taken together, these findings suggest that the relationship between adiposity and iron status is not adequately captured by BMI alone and may be partly mediated by inflammatory processes that are not fully reflected in conventional iron biomarkers. Elevated AGP concentrations were independently associated with increased odds of IDA, supporting a role for sustained low-grade inflammation in disrupting iron metabolism beyond what conventional acute-phase markers capture. Menstrual iron loss also emerged as an important determinant of anaemia and IDA, underscoring the combined influence of inflammatory burden and physiological iron demand on iron status among women of reproductive age. Longitudinal studies incorporating a comprehensive panel of iron biomarkers, including serum iron, sTfR, and transferrin saturation, are needed to clarify the mechanisms linking adiposity, inflammation, and iron regulation and to inform more effective screening and intervention strategies in the Indonesian context.

## Figures and Tables

**Table 1:** Sociodemographic, anthropometric, and menstrual history of participants.

Variable	Group		*p*-Value ^†^
	Normal BMI (*n* = 166)	Overweight/Obesity (*n* = 174)	
Age (years) [med, IQR]	21.00 [2.0]	21.00 [2.0]	0.187
Residential place (*n*, %)			0.203
Living with parents	48 [28.9]	62 [35.6]	
Living without parents	118 [71.1]	112 [64.4]	
Education level (*n*, %)			0.142
High school graduate	150 [90.4]	148 [85.1]	
University graduate	16 [9.6]	26 [14.9]	
Working status (*n*, %)			0.097
Working	11 [6.6]	21 [12.1]	
Not working	155 [93.4]	153 [87.9]	
Monthly income (*n*, %)			0.009 *
<150 USD	135 [81.3]	120 [69.0]	
≥150 USD	31 [18.7]	54 [31.0]	
Anthropometric measure			
BMI (kg/m^2^) [med, IQR]	20.13 [1.9]	25.86 [4.8]	<0.001 *
Body fat (%) [med, IQR]	28.30 [4.9]	34.3 [4.9]	<0.001 *
Waist circumference (cm) [med, IQR]	67.5 [6.2]	80.0 [10.5]	<0.001 *
General morbidity (*n*, %)			0.460
Healthy	46 [27.7]	42 [24.1]	
Not healthy	120 [72.3]	132 [75.9]	
Physical activity level (METs) [med, IQR]	372.00 [648.0]	672.50 [867.0]	<0.001 *
Physical activity (*n*, %)			0.004 *
Low	114 [68.7]	92 [52.9]	
Moderate-vigorous	52 [31.3]	82 [47.1]	
Age of menarche (years) [mean, SD]	12.6 [1.4]	12.2 [1.2]	0.005 *
Estimated menstrual blood loss (mL) ^‡^ [med, IQR]	150.00 [105.0]	135.00 [96.0]	0.223
Estimated iron loss (mg) ^§^ [med, IQR]	60.00 [42.0]	54.00 [39.0]	0.223

Note: Continuous variables: mean ± SD or median [IQR]; Categorical variables: n (%); * Two-sided significance at α < 0.05; ^†^ Test: *t*-test/Mann–Whitney U for continuous, Chi-square/Fisher’s exact for categorical; *p*-values indicate differences across BMI group; ^‡^ Estimated menstrual blood loss = duration × flow × product usage × 0.4 mg/mL; ^§^ Estimated iron loss = menstrual blood loss × 0.4 [30,31,32,33].

**Table 2:** Iron status and inflammatory biomarkers of the normal-weight group and the group with overweight/obesity.

Variable	Normal BMI (*n* = 166)	Overweight/Obesity (*n* = 174)	*p*-Value ^†^
Haemoglobin (g/dL) [med, IQR]	12.60 [1.6]	12.80 (1.3)	0.146
Adjusted Serum Ferritin (µg/L) *** (mean, SD)	17.78 ± 17.6	22.39 ± 21.7	0.033 *
C-reactive protein (CRP) (mg/L) [med, IQR]	0.47 [0.5]	0.51 [0.6]	0.205
Alpha-1-acid Glycoprotein (AGP, g/L) [med, IQR]	0.88 [0.1]	0.88 [0.1]	0.390
Hepcidin (nmol/L) (mean, SD)	28.84 ± 20.59	29.51 ± 20.39	0.283

*** BRINDA-adjusted ferritin values [14]; ^†^ *t*-test for normally distributed data and Mann–Whitney U for not normally distributed data; * Two-sided Significance at α < 0.05.

**Table 3:** Multivariable logistic regression models for iron deficiency (ID).

Predictor	Crude	Adjusted OR (95% CI)		
	OR (95% CI)	Model I ^a^	Model II ^b^	Model III ^c^
BMI category				
Normal	1.00 (ref)	1.00 (ref)	1.00 (ref)	1.00 (ref)
Overweight/Obesity	0.64 (0.41–1.01)	0.64 (0.40–1.01)	0.58 (0.36–0.95) *	0.82 (0.43–1.56)
Education level				
University graduate		1.00 (ref)	1.00 (ref)	1.00 (ref)
High-school graduate		1.35 (0.48–3.78)	1.63 (0.55–4.88)	1.69 (0.56–5.07)
Employment status				
Working		1.00 (ref)	1.00 (ref)	1.00 (ref)
Not working		2.06 (0.67–6.30)	1.77 (0.55–5.72)	1.64 (0.50–5.34)
Monthly income				
≥150 USD		1.00 (ref)	1.00 (ref)	1.00 (ref)
<150 USD		1.62 (0.90–2.89)	1.55 (0.85–2.82)	1.51 (0.75–3.05)
Animal-source food intake				
≥3 portions/day			1.00 (ref)	1.00 (ref)
<3 portions/day			1.32 (0.81–2.16)	1.27 (0.77–2.09)
Vitamin C intake category				
≥86 mg			1.00 (ref)	1.00 (ref)
<35 mg			1.49 (0.75–2.98)	1.51 (0.75–3.05)
35–86 mg			0.85 (0.44–1.64)	0.88 (0.45–1.71)
Dietary diversity				
Adequate			1.00 (ref)	1.00 (ref)
Inadequate			1.50 (0.92–2.44)	1.56 (0.95–2.55)
Physical activity level				
Medium-high intensity			1.00 (ref)	1.00 (ref)
Low intensity			1.65 (1.01–2.71) *	1.70 (1.00–2.81)
Tea and coffee intake (mL/d)			1.00 (0.99–1.00)	1.00 (0.99–1.00)
Iron intake (mg/d)			1.00 (0.99–1.02)	1.00 (0.98–1.02)
Fibre intake (g/d)			1.04 (0.98–1.11)	1.04 (0.98–1.11)
Body fat (%)				0.93 (0.86–0.99) *
Menstrual iron loss (mg)				1.01 (0.99–1.01)
Hepcidin concentrations (nmol/L)				0.99 (0.98–1.01)
R^2^	0.015	0.044	0.102	0.122

* *p* value < 0.05; ^a^ Model I adjusted for socio-demographic (educational level, working status, monthly income), ^b^ Model II adjusted for Model I + dietary behaviour (tea and coffee intake, iron intake, fibre intake), ^c^ Model III adjusted for Model I + Model II + physiological factor, body composition and iron biomarkers.

**Table 4:** Multivariable logistic regression models for anaemia.

Predictor	Crude	Adjusted OR (95% CI)	
	OR (95% CI)	Model I ^a^	Model II ^b^
BMI category			
Normal	1.00 (ref)	1.00 (ref)	1.00 (ref)
Overweight/Obesity	0.71 (0.44–1.16)	0.75 (0.46–1.23)	1.07 (0.54–2.11)
Iron intake (mg/d)		1.01 (0.99–1.03)	1.01 (0.99–1.03)
Zinc intake			
≥6.70 mg/day		1.00 (ref)	1.00 (ref)
4.67–6.70 mg/day		1.78 (0.77–4.16)	2.17 (0.91–5.17)
<4.67 mg/day		0.77 (0.36–1.65)	0.86 (0.39–1.87)
Phytate intake category			
<1200 mg/day		1.00 (ref)	1.00 (ref)
≥1200 mg/day		1.02 (0.51–2.07)	1.10 (0.54–2.27)
Body fat category			
<30%			1.00 (ref)
≥30%			0.59 (0.29–1.18)
Menstrual iron loss (mg)			1.01 (1.00–1.02) *
Inflammation-adjusted ferritin (µg/L)			0.63 (0.26–1.53)
Hepcidin concentrations (nmol/L)			1.01 (0.99–1.02)
R^2^	0.008	0.046	0.098

* *p* value < 0.05; ^a^ Model I adjusted for nutrient intake (iron, zinc, phytate), ^b^ Model II adjusted for Model I + body fat percentage, estimated iron loss, adjusted ferritin, and hepcidin level.

**Table 5:** Multivariable logistic regression models for iron deficiency anaemia (IDA).

Predictor	Crude	Adjusted OR (95% CI)		
	OR (95% CI)	Model I ^a^	Model II ^b^	Model III ^c^
BMI category				
Normal	1.00 (ref)	1.00 (ref)	1.00 (ref)	1.00 (ref)
Overweight/Obesity	0.56 (0.27–1.15)	0.46 (0.21–1.02)	0.58 (0.18–1.87)	0.65 (0.18–2.32)
Zinc intake				
≥6.70 mg/day		1.00 (ref)	1.00 (ref)	1.00 (ref)
4.67–6.70 mg/day		2.68 (0.67–10.79)	3.10 (0.77–12.51)	4.02 (0.94–17.24)
<4.67 mg/day		0.73 (0.19–2.79)	0.84 (0.22–3.23)	0.92 (0.23–3.71)
Phytate intake category				
<1200 mg/day		1.00 (ref)	1.00 (ref)	1.00 (ref)
≥1200 mg/day		2.04 (0.57–7.23)	2.15 (0.61–7.61)	2.18 (0.59–8.11)
Animal-source food intake				
≥3 portions/day		1.00 (ref)	1.00 (ref)	1.00 (ref)
<3 portions/day		1.82 (0.82–4.02)	1.79 (0.79–4.05)	1.83 (0.76–4.41)
Physical activity level				
Medium-high intensity		1.00 (ref)	1.00 (ref)	1.00 (ref)
Low intensity		2.34 (1.05–5.22) *	2.24 (0.99–5.03)	2.06 (0.88–4.81)
Iron intake (mg/d)		1.02 (0.99–1.03)	1.02 (1.00–1.04) *	1.02 (1.00–1.04) *
Vitamin C intake (mg/d)		1.00 (0.99–1.00)	1.00 (0.99–1.00)	1.00 (0.99–1.01)
Fibre intake (g/d)		1.09 (0.98–1.20)	1.09 (0.99–1.21)	1.09 (0.97–1.20)
Tea and coffee intake (ml/d)		1.00 (1.00–1.01)	1.00 (0.99–1.01)	1.00 (0.99–1.01)
Dietary diversity score		1.18 (0.95–1.47)	1.14 (0.89–1.43)	1.09 (0.84–1.41)
Body fat percentage (%)			0.89 (0.78–1.02)	0.86 (0.75–0.99) *
Waist circumference (cm)			1.04 (0.97–1.11)	1.05 (0.98–1.13)
Educational background			2.54 (0.89–7.27)	0.39 (0.13–1.23)
Age of menarche (years)				1.31 (0.96–1.78)
Estimated iron loss (mg)				1.02 (1.00–1.03) *
AGP concentrations (g/L)				8.77 (1.01–77.34) *
Hepcidin concentrations (nmol/L)				0.99 (0.97–1.02)
R^2^	0.016	0.166	0.197	0.265

* *p* value < 0.05; ^a^ Model I adjusted for dietary intake (animal-source of food intake, iron, zinc, vitamin C, fibre, phytate), dietary diversity, and physical activity, ^b^ Model II adjusted for Model I + body fat, waist circumference and educational background, ^c^ Model III adjusted for Model I + Model II + physiological factors (age of menarche, iron loss), and AGP concentrations.

## Data Availability

The data presented in this study are available on request from the corresponding author upon reasonable request due to ethical and privacy restrictions.

## Supplementary Materials

The following supporting information can be downloaded at: https://www.mdpi.com/article/10.3390/nu18162583/s1. Table S1: Differences in dietary behaviours between the normal-weight group and the group with overweight/obesity; Table S2: Binary logistic analysis for predictors of Iron Deficiency (ID), Anaemia, and Iron Deficiency Anaemia (IDA). References [54,55,56] are cited in the Supplementary Materials.

## Abbreviations

The following abbreviations are used in this manuscript:

ID	Iron deficiency
IDA	Iron deficiency anaemia
NW	Normal weight
OWT	Overweight
OB	Obesity/Obese
SQFFQ	Semi-quantitative food frequency questionnaire
MET	Metabolic equivalents of task
CRP	C-reactive protein
AGP	α_1_-acid glycoprotein
WRA	Women of reproductive age
LMICs	Low-and middle-income countries
