# Supplementary material for: Overweight/Obesity and Body Fat Percentage in Relation to Iron Status Among Indonesian Women of Reproductive Age: Evidence from a Case–Control Study
Source: Nutrients. 2026 Aug 7;18(16):2583. doi: 10.3390/nu18162583 (PMC13516781; doi:10.3390/nu18162583)
Supplement: Supplementary file 1 [file nutrients-18-02583-s001.zip › Supplementary Table S1.pdf]

Supplementary Table S1. Differences in dietary behaviours between the normal-weight group and the group with overweight/obesity

Variable	Group		*P-value*[ƚ]
	**Normal BMI (n = 166)**	**Overweight/Obese (n = 174)**	
Energy (kcal) [med, IQR]	1573.75 [721.1]	1552.20 [782.0]	0.957
Energy intake category[¦¦] (n, %)			
<2250 kcal/d	147 (88.6)	148 (86.5)	0.623
≥2250 kcal/d	19 (11.4)	23 (13.5)	
Iron (mg) [med, IQR]	14.60 [14.3]	14.70 [10.1]	0.928
Iron intake category (n, %)			
Tertile 1 (<11.5 mg/d)	59 (35.5)	51 (29.3)	0.040[*]
Tertile 2 (11.5 – 18.67 mg/d)	45 (27.1)	71 (40.8)	
Tertile 3 (>18.67 mg/d)	62 (37.3)	52 (29.9)	
Vitamin C (mg) (mean ± SD)	89.43 [104.9]	101.03 [133.8]	0.374
Vitamin C intake category (n, %)			
Tertile 1 (<35.53 mg/d)	60 (36.2)	53 (30.4)	0.398
Tertile 2 (35.53 – 86.15 mg/d)	53 (31.9)	61 (35.1)	
Tertile 3 (≥86.15 mg/d)	53 (31.9)	60 (34.5)	
Zinc (mg) [med, IQR]	5.50 [4.5]	5.60 [3.1]	0.682
Zinc intake category (n, %)			
Tertile 1 (<4.66 mg/d)	59 (35.5)	54 (31.0)	0.161
Tertile 2 (4.66 – 6.70 mg/d)	45 (27.1)	64 (36.8)	
Tertile 3 (≥6.70 mg/d)	62 (37.3)	56 (32.2)	
Calcium (mg) (mean ± SD)	530.10 (454.5)	484.60 (354.9)	0.978
Calcium intake category[¶] (n, %)			
<501.19 mg/d	77 (46.4)	94 (54.0)	0.193
≥501.19 mg/d	89 (53.6)	80 (46.0)	
Phytate (mg) [med, IQR]	1129.00 [1028.0]	1088.45 [763.6]	0.517
Phytate intake category[**] (n, %)			
<1200 mg/d	91 (54.8)	103 (59.2)	0.444
≥1200 mg/d	75 (45.2)	71 (40.8)	
Fibre (gr) (mean ± SD)	6.03 (5.3)	5.92 (5.5)	0.959
Fibre intake category[¶] (n, %)			
<4.47 g/d	87 (52.4)	89 (51.1)	0.829
≥4.47 g/d	79 (47.6)	85 (48.9)	
Daily intake of Animal Source of Foods[¦¦] (n, %)			
<3 portion/day	103 (62.0)	105 (60.3)	0.824
≥3 portions/day	63 (38.0)	69 (39.7)	
Frequency of tea intake			
Daily (≥1x/day)	5 (3.0)	3 (1.7)	0.494
Occasionally-rarely (1-6x/week)	161 (97.0)	171 (98.3)	
Frequency of coffee intake			
Daily (≥1x/day)	4 (2.4)	11 (6.3)	0.112
Occasionally-rarely (1-6x/week)	162 (97.6)	163 (93.7)	
Tea and coffee intake (ml/day)	16.67 (67.3)	17.34 (66.7)	0.894
Dietary diversity score (mean ± SD)	4.62 (1.7)	4.72 (1.7)	0.603
Dietary diversity (n, %)			
Adequate	86 (51.8)	93 (54.1)	0.664
Inadequate	80 (48.2)	79 (45.9)	

[ƚ]Test: T-test/Mann-Whitney U for continuous, Chi-square/Fisher's exact for categorical; *p*-values indicate differences across BMI groups.

[¦¦]Based on the Indonesian dietary guidelines [1] and the Indonesian Recommended Dietary Allowance [2]; [¶]mean intake; [**]Acceptable Daily Intake [3]

[8]Two-sided significance at $\alpha < 0.05$

References

1. Indonesia Ministry of Health. Peraturan Menteri Kesehatan Nomor 41 Tahun 2014 tentang Pedoman Gizi Seimbang. Indonesia Ministry of Health: Jakarta, 2014.
2. Indonesia Ministry of Health. Peraturan Menteri Kesehatan Republik Indonesia Nomor 28 Tahun 2019 tentang Angka Kecukupan Gizi yang Dianjurkan untuk Masyarakat Indonesia. Indonesia Ministry of Health: 2019.
3. Gibson, R.S.; Raboy, V.; King, J.C. Implications of phytate in plant-based foods for iron and zinc bioavailability, setting dietary requirements, and formulating programs and policies. *Nutrition reviews* **2018**, *76*, 793-804.
