# Supplementary material for: Overweight/Obesity and Body Fat Percentage in Relation to Iron Status Among Indonesian Women of Reproductive Age: Evidence from a Case–Control Study
Source: Nutrients. 2026 Aug 7;18(16):2583. doi: 10.3390/nu18162583 (PMC13516781; doi:10.3390/nu18162583)
Supplement: Supplementary file 1 [file nutrients-18-02583-s001.zip › Supplementary Table S2.pdf]

Supplementary Table S2. Binary logistic analysis for predictors of Iron Deficiency (ID), Anaemia, and Iron Deficiency Anaemia (IDA)

Variable	Iron Deficiency (ID)[a]		Anaemia[b]		Iron Deficiency Anaemia (IDA)[c]	
	**Crude OR (95% CI)**	***p-value***	**Crude OR (95% CI)**	***p-value***	**Crude OR (95% CI)**	***p-value***
BMI category						
Normal weight	1.00	0.056	1.00	0.171	1.00	0.115
Overweight/Obese	0.64 (0.41 – 1.01)		0.71 (0.44 – 1.16)		0.56 (0.27 – 1.15)	
BMI category						
Normal weight	1.00		1.00		1.00	
Overweight	0.52 (0.27 – 0.98)	0.031*	0.71 (0.36 – 1.40)	0.323	0.34 (0.10 – 1.19)	0.089
Obese	0.73 (0.44 – 1.21)	0.225	0.78 (0.45 – 1.36)	0.389	0.69 (0.31 – 1.53)	0.361
BMI (kg/m^2)	0.96 (0.91 – 1.01)	0.109	1.03 (0.96 – 1.06)	0.611	0.97 (0.89 – 1.05)	0.453
Body fat (%)						
<30%	1.00		1.00		1.00	
≥30%	0.63 (0.40 - 1.00)	0.051	0.59 (0.36 - 0.98)	0.041*	0.50 (0.25 - 1.02)	0.057
Body fat (%)	0.95 (0.91 – 0.99)	0.034*	0.98 (0.94 – 1.04)	0.602	0.94 (0.90 – 1.01)	0.084
Waist circumference category						
<80 cm	1.00		1.00		1.00	
≥80 cm	0.80 (0.48 - 1.34)	0.398	1.11 (0.64 - 1.91)	0.709	0.78 (0.34 - 1.79)	0.554
Waist circumference (cm)	1.00 (0.99 – 1.01)	0.434	1.00 (0.97 – 1.01)	0.290	1.01 (0.99 – 1.01)	0.153
Age (years)	1.04 (0.91 – 1.19)	0.550	0.99 (0.86 – 1.15)	0.943	1.02 (0.83 – 1.26)	0.837
Residential place						
Without parents	1.00		1.00		1.00	
With parents	0.97 (0.64 – 1.66)	0.908	0.99 (0.59 – 1.67)	0.969	0.99 (0.47 – 2.13)	1.000
Education level						
University graduate	1.00		1.00		1.00	
High-school graduate	1.71 (0.89 – 3.29)	0.107	1.19 (0.58 – 2.44)	0.636	2.01 (0.81 – 4.95)	0.130
Working status						
Working	1.00		1.00		1.00	
Not working	2.08 (1.00 – 4.33)	0.046*	1.15 (0.51 – 2.60)	0.730	1.78 (0.64 – 4.98)	0.271
Monthly income						
≥150 USD	1.00		1.00		1.00	
<150 USD	1.44 (0.84 – 2.45)	0.188	1.38 (0.77 – 2.49)	0.283	1.32 (0.55 – 3.15)	0.532
General morbidity						
No current morbidity	1.00		1.00		1.00	
Present morbidity	1.14 (0.69 – 1.89)	0.606	1.13 (0.64 – 1.99)	0.667	1.42 (0.66 – 3.05)	0.366
Animal-source food intake (portion)	1.07 (0.88 – 1.30)	0.524	1.11 (0.90 – 1.37)	0.323	1.29 (0.96 – 1.75)	0.091

Animal-source food intake (category)						
≥3 portions/day	1.00		1.00		1.00	
<3 portion/day	1.39 (0.88 – 2.19)	0.162	1.15 (0.70 – 1.89)	0.571	1.66 (0.82 – 3.38)	0.162
Frequency of iron inhibitor consumption: Tea						
Occasionally-rarely (1-6x/week)	1.00		1.00		1.00	
Daily (≥1x/day)	1.16 (0.27 – 4.95)	0.838	0.97 (0.19 – 4.89)	0.969	0.11 (0.01 – 1.00)	0.999
Frequency of iron inhibitor consumption: Coffee						
Occasionally-rarely (1-6x/week)	1.00		1.00		1.00	
Daily (≥1x/day)	0.69 (0.22 – 2.22)	0.536	1.06 (0.33 - 3.42)	0.922	1.41 (0.30 - 6.52)	0.661
Tea and coffee intake (ml/day)	1.00 (0.99 – 1.00)	0.207	1.00 (0.99 – 1.00)	0.553	1.00 (0.99 – 1.01)	0.183
Energy (kcal/day)	1.00 (1.00 – 1.00)	0.875	1.00 (1.00 – 1.00)	0.981	1.00 (1.00 – 1.00)	0.534
Energy intake category						
<2250 kcal/day	1.00		1.00		1.00	
≥2250 kcal/day	1.10 (0.56 – 2.16)	0.782	1.55 (0.69 – 3.50)	0.287	1.08 (0.36 – 3.22)	0.897
Iron (mg/day)	1.01 (0.99 – 1.02)	0.192	1.02 (0.99 – 1.02)	0.151	1.02 (1.00 – 1.03)	0.045*
Iron intake category						
Tertile 1 (< 11.5 mg/d)	1.00		1.00		1.00	
Tertile 2 (11.5 – 18.67 mg/d)	0.88 (0.50 – 1.52)	0.640	0.66 (0.26 – 1.21)	0.181	0.76 (0.32 – 1.85)	0.549
Tertile 3 (>18.67 mg/d)	0.79 (0.62 – 1.87)	0.786	0.88 (0.49 – 1.58)	0.672	0.97 (0.42 – 2.26)	0.944
Vitamin C (mg/day)	1.00 (0.99 – 1.00)	0.161	1.00 (0.99 – 1.00)	0.286	1.00 (0.99 – 1.00)	0.131
Vitamin C intake category						
Tertile 3 (>87.10 mg/d)	1.00		1.00		1.00	
Tertile 1 (<35.48 mg/d)	0.67 (0.38 – 1.17)	0.158	0.77 (0.42 – 1.41)	0.393	0.89 (0.39 – 2.06)	0.793
Tertile 2 (35.48 – 87.10 mg/d)	0.99 (0.57 – 1.72)	0.973	0.80 (0.44 – 1.44)	0.454	0.60 (0.24 – 1.46)	0.257
Zinc (mg/day)	1.01 (0.97 – 1.04)	0.772	0.99 (0.95 – 1.04)	0.775	1.00 (0.94 – 1.06)	0.989
Zinc intake category (n, %)						
Tertile 1 (<4.67 mg/d)	1.00		1.00		1.00	
Tertile 2 (4.67 – 6.70 mg/d)	0.90 (0.52 – 1.56)	0.712	0.44 (0.23 – 0.81)	0.009*	0.29 (0.10 – 0.82)	0.020*
Tertile 3 (>6.70 mg/d)	1.19 (0.69 – 2.06)	0.535	1.18 (0.66 – 2.09)	0.579	0.96 (0.44 – 2.10)	0.924
Calcium (mg/day)	1.00 (1.00 – 1.00)	0.841	1.00 (0.99 – 1.00)	0.842	1.00 (0.99 – 1.00)	0.648
Calcium intake category (n, %)						
<501.19 mg/d	1.00		1.00		1.00	
≥501.19 mg/d	1.07 (0.69 - 1.68)	0.759	0.77 (0.47 - 1.25)	0.292	1.01 (0.50 - 2.06)	0.971
Phytate (mg/day)	1.00 (1.00 – 1.00)	0.786	1.00 (1.00 – 1.00)	0.320	1.00 (1.00 – 1.00)	0.591
Phytate intake category (n, %)						

<1200 mg/day	1.00		1.00		1.00	
≥1200 mg/day	1.25 (0.70 – 2.23)	0.488	1.45 (0.79 - 2.66)	0.236	1.80 (0.79 - 4.08)	0.160
Fibre (g/day)	1.03 (0.99 – 1.08)	0.101	1.02 (0.98 – 1.07)	0.287	1.06 (1.01 – 1.11)	0.031*
Fibre intake category (n, %)						
<4.47 g/d	1.00		1.00		1.00	
≥4.47 g/d	1.06 (0.68 – 1.65)	0.811	1.00 (0.62 – 1.63)	0.993	1.20 (0.59 – 2.45)	0.613
Dietary diversity score	1.10 (0.97 – 1.26)	0.152	1.06 (0.92 – 1.22)	0.452	1.20 (0.97 – 1.48)	0.096
Dietary Diversity						
Adequate	1.00		1.00		1.00	
Inadequate	1.55 (0.98 – 2.44)	0.059	1.04 (0.64 – 1.70)	0.864	1.47 (0.71 – 3.05)	0.296
Physical activity level (METs)	1.00 (1.00 – 1.00)	0.315	1.00 (1.00 – 1.00)	0.608	1.00 (1.00 – 1.00)	0.325
Physical Activity Level						
Medium-high intensity	1.00		1.00		1.00	
Low intensity	1.33 (0.85 – 2.10)	0.217	1.05 (0.64 – 1.72)	0.856	1.62 (0.79 – 3.29)	0.186
Age of menarche (years)	1.10 (0.93 – 1.30)	0.270	1.10 (0.92 – 1.32)	0.310	1.26 (0.97 – 1.63)	0.079
Estimated menstrual blood loss (ml)	1.00 (0.99 – 1.01)	0.139	1.00 (1.00 – 1.01)	**0.005***	1.00 (1.00 – 1.01)	0.015*
Estimated menstrual iron loss (mg)	1.00 (0.99 – 1.01)	0.139	1.01 (1.00 – 1.02)	**0.005***	1.01 (1.00 – 1.02)	0.015*
Haemoglobin levels (g/dL)	0.91 (0.80 – 1.02)	0.097	-	-	-	
Adjusted-ferritin levels (μg/l)†	-	-	0.59 (0.25 – 1.38)	0.226	-	
C-reactive protein (CRP) levels (mg/L)	0.86 (0.64 – 1.16)	0.322	1.00 (0.98 – 1.03)	0.801	0.94 (0.796 – 1.12)	0.506
CRP category						
Normal CRP (≤5 mg/L)	1.00		1.00		1.00	
High CRP (>5 mg/L)	1.58 (0.74 – 3.36)	0.238	1.16 (0.55 – 2.45)	0.691	1.38 (0.40 – 4.74)	0.611
Alpha-1-acid Glycoprotein (AGP) levels (g/L)	0.53 (0.17 – 1.61)	0.263	1.88 (0.55 – 6.51)	0.315	3.07 (0.50 – 18.96)	0.227
AGP category						
Normal AGP (≤1 g/L)	1.00		1.00		1.00	
High AGP (>1 g/L)	1.16 (0.56 – 2.39)	0.690	1.18 (0.54 – 2.58)	0.670	2.05 (0.78 – 5.35)	0.144
Hepcidin concentrations (nmol/L)	0.99 (0.98 – 1.00)	0.126	1.00 (0.99 – 1.02)	0.632	0.99 (0.975 – 1.02)	0.670
Hepcidin						
<median (<29.42 nmol/L)	1.00		1.00		1.00	
≥median (≥29.42 nmol/L)	0.87 (0.54 – 1.39)	0.552	1.14 (0.68 – 1.93)	0.614	1.05 (0.49 – 2.23)	0.908

[a]ID is defined based on adjusted serum ferritin values <15 μg/L; [b]Anaemia is defined based on haemoglobin values <12 μg/L; [c]IDA is defined based on adjusted serum ferritin values <15 μg/L and haemoglobin values <12 μg/L; †BRINDA-adjusted ferritin values
